# Long sleep duration is associated with cognitive frailty among older community-dwelling adults: results from West China Health and Aging Trend study

**DOI:** 10.1186/s12877-021-02455-9

**Published:** 2021-10-27

**Authors:** Yunli Zhao, Ying Lu, Wanyu Zhao, Yanyan Wang, Meiling Ge, Lixing Zhou, Jirong Yue, Birong Dong, Qiukui Hao

**Affiliations:** 1 National Clinical Research Center for Geriatrics, West China Hospital, Sichuan University, No.37 Guo Xue Xiang, Chengdu, China; 2 Center of Gerontology and Geriatrics, West China Hospital, Sichuan University, No.37 GuoXueXiang, Chengdu, 610041 Sichuan China; 3 National Clinical Research Center for Geriatrics and Department of General Practice, State Key Laboratory of Biotherapy, West China Hospital, Sichuan University, and Collaborative Innovation Center of Biotherapy, Chengdu, China

**Keywords:** Cognitive frailty, Older adults, Sleep duration

## Abstract

**Objective:**

To investigate the association between sleep duration and cognitive frailty among older adults dwelling in western China.

**Methods:**

We used the baseline data from West China Health and Aging Trend (WCHAT) study. Sleep duration was classified as short sleep duration (< 6 h), normal sleep duration (6–8 h) and long sleep duration (≥ 9 h). Fried frailty criteria and Short Portable Mental Status Questionnaire were used to measure cognitive frailty. Multinomial logistic regression was conducted to estimate odds ratio (OR) and 95% confidence interval (CI).

**Results:**

A total of 4093 older adults (age = 67.8 ± 5.9 years, 1708 males and 2385 females) were included in the analysis. The prevalence of cognitive frailty was 11.8% among older adults in western China. Approximately 11.9% participants had short sleep duration (< 6 h); 22.2% had a long sleep duration (≥ 9 h). After adjusting for covariates, only long sleep duration was significantly associated with high risk of cognitive frailty (OR = 2.07, 95%CI = 1.60–2.68, *P* <  0.001) in western China older adults compared to normal sleep duration.

**Conclusions:**

Long sleep duration was significantly related to cognitive frailty in older adults. Intervention for long sleep duration may be helpful to prevent cognitive frailty.

**Trial registration:**

Chinese Clinical Trial Registry: ChiCTR1800018895.

## Introduction

Physical frailty and cognitive impairment are two common and pervasive medical conditions among older adults. The prevalence of physical frailty in older adults ranged from 4.0 to 75.6% [1, 2] and the prevalence of cognitive impairment was estimated to be 3–42% [3]. Recent studies suggested that 70% of physically frail older adults coexisted cognitive impairment [4], and half of older adults with cognitive impairment were also physical frail [5]. Both physical frailty and cognitive impairment were associated with a range of deleterious outcomes in older adults such as lower quality of life (QOL) [6, 7], increased use of health care services, risk of morbidity and mortality [8, 9]. Based on the evidence revealing the relationship between physical frailty and cognitive impairment, the concept of “cognitive frailty” was first proposed in 2013 to describe a clinical condition characterized by the co-existing physical frailty and cognitive impairment but no dementia (CIND) [10]. Previous studies have demonstrated that physical frailty was a significant predictor of Alzheimer disease (AD) among older adults [11]. Compared with older adults with physical frailty or cognitive impairment alone, those with cognitive frailty were considered to have a higher risk of AD, disability and mortality [12, 13]. Therefore, public health efforts are deserved to identify the risk factors of cognitive frailty among older adults.

Sleep, a biobehavioral phenomenon regulated by circadian, homeostatic, and neurohormonal processes [14], is critical for maintaining physical performance and cognitive function [15]. Sleep disorder is very common, and a decrease in total sleep duration often occurs among older adults [16]. Previous studies have found that physical frailty and cognitive impairment was significantly associated with sleep duration among older adults, respectively [17, 18]. However, to our knowledge, the association between sleep duration and cognitive frailty in older adults has not been investigated.

To fill this gap, the aims of this study are as follows: 1) to determine the prevalence of cognitive frailty among community-dwelling older adults in the western China; 2) to explore the association between cognitive frailty and sleep duration.

## Methods

### Study design and sample selection

Data were based on the baseline of West China Health and Aging Trend (WCHAT) study, a population-based longitudinal study of aging and health of community-dwelling Chinese aged 50 and older in western China [19]. The cohort study began in 2018 and was approved by the Ethics Committee of West China Hospital, Sichuan University (reference: 2017–445) and was conducted in accordance with the 2013 version of the Declaration of Helsinki. The study registered at the Chinese Clinical Trial Registry under number ChiCTR1800018895. Trained interviewers collected the baseline data through face-to-face interviews and physical examination. A written informed consent was obtained from all participants (or their legal proxies for those who were unable to sign their names).

A total of 7536 participants from 18 ethnic groups in Sichuan, Yunnan, Guizhou and Xinjiang province were included in the WCHAT study. To better analyze the association between sleep duration and cognitive frailty, we only included the participants aged 60 and older with relevant data in the present study. We eventually included 4093 participants in the current analysis, after excluding 3022 participants under 60 years old, 370 with missing data on physical frailty phenotype, 41 with missing data on cognitive function, 3 diagnosed with dementia, and 7 with missing data on the sleep duration.

### Physical frailty assessment

The modified Fried frailty phenotype was used to assess physical frailty in this study [20]. The detailed criteria include:

- Shrinking: self-reported unintentional weight loss of 4.5 kg in last year or body mass index (BMI) < 18.5 kg/m2.
- Weakness: maximum grip strength in dominant hand ≤20% of the population distribution, adjusted for gender and body mass index (BMI).
- Exhaustion: self-reported lack of energy (energy score was no more than 3, when 10 represents the most powerful condition); or self-reported excessively fatigue or weak for most of the time.
- Slowness: walking time/4-m ≤ 20% of the population distribution, adjusted for gender and height.

Low physical activity level: energy consumption (kcal/week) spend on commonly performed physical activities ≤20% of the population distribution, adjusted for gender. Physical activities were measured by using a validated China Leisure Time Physical Activity Questionnaire (CLTPAQ) [21], which was a modified version of the Minnesota Leisure Time Physical Activity Questionnaire (MLTPAQ) [22] based on the Chinese lifestyle and cultural background.

According to previously established cut-points [20], we specified that a phenotype of physical frailty was indicated by the presence of three or more of the items, pre-physical frailty was indicated by the presence of one or two of the items and robust was the absence of all items.

### Cognitive assessment

We used the 10-point Short Portable Mental Status Questionnaire (SPMSQ) to assess cognitive function of the participants [23]. A score greater than 4 in participants with primary school education and lower, or a score of greater than 2 in participants with high school education and higher are defined as cognitive impairment [24, 25].

### Definition of cognitive frailty

As in previous studies [26, 27], we divided our participants into five groups according to physical frailty status and cognitive function: 1) robust group, robust older adults who had no physical frailty and cognitive impairment, 2) pre-physical frailty group, physically pre-frail older adults without cognitive impairment, 3) physical frailty group, physically frail older adults without cognitive impairment, 4) cognitive impairment group, non-physically pre-frail/frail older adults but with cognitive impairment, and 5) cognitive frailty group, physically pre-frailty/frailty older adults with cognitive impairment.

### Sleep duration

We used the following question, “During the past month, how many hours of actual sleep did you get at night (average hours for one night)” to collect the sleep duration from the participants. Based on the recommendations of sleep duration from the American Academy of Sleep Medicine [28], we divided our participants into three groups. Short sleep duration was < 6 h, normal sleep duration was 6–8 h, and long sleep duration ≥9 h, and normal sleep duration was chosen as the referent categories.

### Covariates

We collected the following information through face-to-face interviews: age, gender, education (illiteracy/primary school/secondary school or advanced), ethnicity (Han/Qiang/Tibetan/other minority ethnic groups) and marital status (married/single), smoking history (yes/no), alcohol drinking history (yes/no) and number of chronic diseases (0/1/≥ 2). We defined smoking and drinking history as a period of regular use of smoking or drinking (i.e. former or current smoker/drinker: drinking/smoking more than half of a week; never smoker/drinker: dinking/smoking less than half of a week). In addition, we evaluated the depression of the participants via 15-item Geriatric Depression Scale (GDS-15) [29]. Depression was defined as GDS-15 score ≥ 8 [29].

### Statistical analysis

Analyses were conducted by using Stata software, version 15.1 (Stata Corp, College Station, TX, USA). Means ± standard deviation (SD) and count (percentage) were used to summarize continuous and categorical variables, respectively. Difference of groups were tested by ANOVA for continuous variables and the chi square test for categorical variables among 5 groups: “robust”, “pre-physical frailty”, “physical frailty”, “cognitive impairment” and “cognitive frailty”. When *p* <  0.05, LSD post-hoc test (continuous variables) or partitions of chi-square (categorical variables) was applied to determine the significant differences among subgroups. Multinomial logistic regression was conducted to find the relationship between cognitive frailty and sleep duration. The multivariable adjusted model included age, gender, ethnicity, education level, marital status, smoking history, drinking history, number of chronic diseases and depression as covariates. Odds ratio (OR) and 95% confidence intervals (CI) were conducted and P<0.05 was used to determine whether the effect was significant.

## Results

**Fig. 1:**
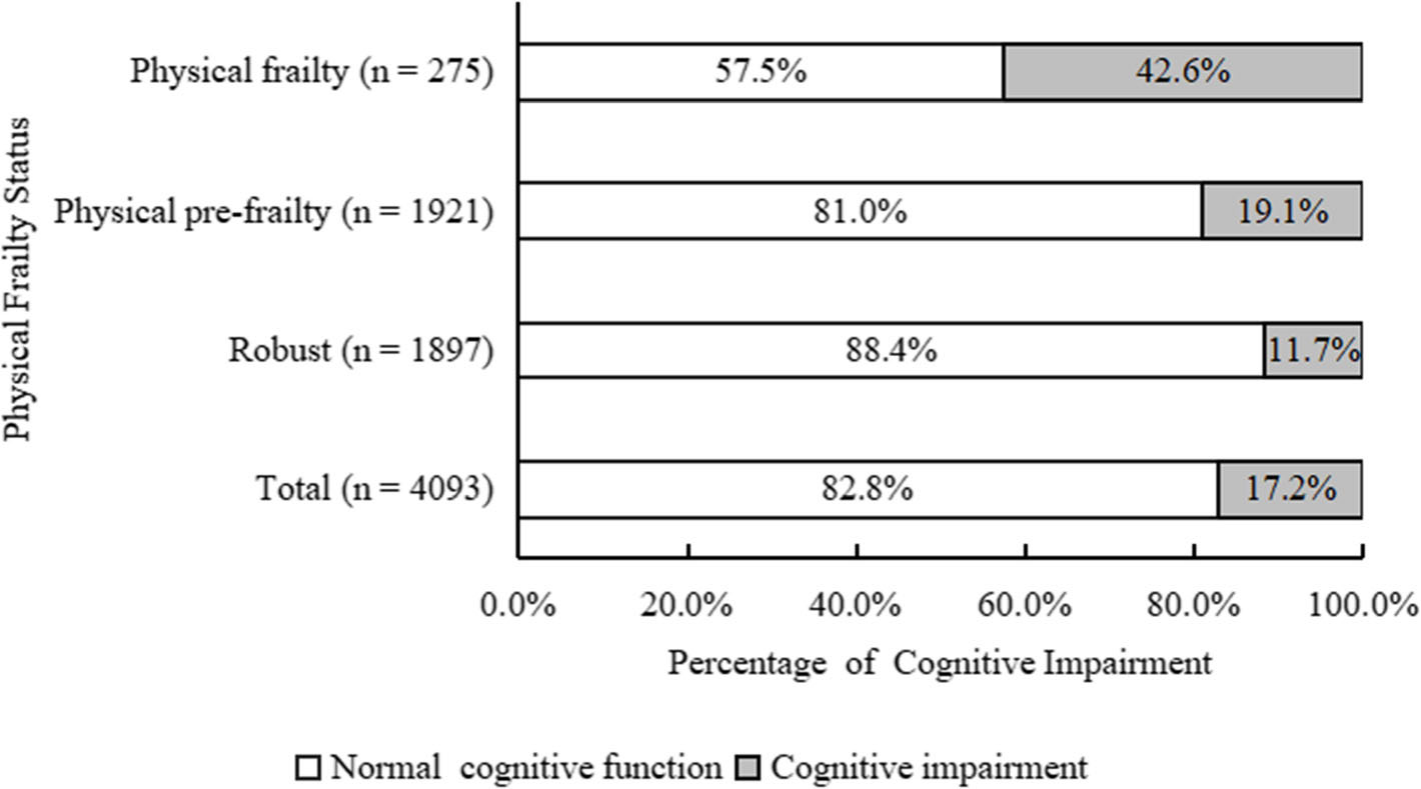
Percentage of participants with cognitive impairment by physical frailty status

Overall, we included 4093 participants (1708 males and 2385 females) in our study. The mean age of the participants was 67.8 ± 5.9 years (ranging from 60 to 95). The percentage of physically frail participants was 6.7% (*n* = 275), while 46.9% (*n* = 1921) were physically pre-frail by the modified Fried frailty phenotype. The prevalence of cognitive impairment was 17.2% (*n* = 704) in our participants. A total of 38.0% (*n* = 1555) participants had pre-physical frailty without cognitive impairment, 3.9% (*n* = 158) had physical frailty without cognitive impairment, 5.4% (*n* = 221) had only cognitive impairment and 11.8% (*n* = 483) had cognitive frailty (Fig. 1). According to the recommendation of sleep duration, 11.9% participants (*n* = 485) had short sleep duration, while 22.2% participants (*n* = 910) had long sleep duration in our study (Table 1).

**Table 1:** Characteristics of the participants according to cognitive frailty status

Characteristics	All subjects (*n* = 4093)	Robust (*n* = 1676)	Physical pre-frailty (*n* = 1555)	Physical frailty (*n* = 158)	Cognitive impairment (*n* = 221)	Cognitive frailty (*n* = 483)	*P*-value
Age (years)^*^	67.8 ± 5.9	66.4 ± 4.9	68.1 ± 6.1^a^	71.9 ± 6.4^b^	67.1 ± 5.6	70.6 ± 6.9^d^	< 0.001
Age (years) (n, %)							< 0.001
60–69	2700 (66.0)	1279 (76.3)	990 (63.7)^a^	60 (38.0)^b^	146 (66.1)^c^	225 (46.6)^d^	
70–79	1205 (29.4)	375 (22.4)	485 (31.2)^a^	74 (47.0)^b^	66 (29.9)^c^	205 (42.4)^d^	
≥ 80	188 (4.6)	22 (1.3)	80 (5.1)^a^	24 (15.2)^b^	9 (4.1)^c^	53 (11.0)^d^	
Gender (n, %)							< 0.001
Males	1708 (41.7)	728 (43.4)	723 (46.5)	83 (52.5)^b^	51 (23.1)^c^	123 (25.5)^d^	
Females	2385 (58.3)	948 (56.6)	832 (53.5)	75 (47.5)^b^	170 (76.9)^c^	360 (74.5)^d^	
Ethnicity (n, %)							< 0.001
Han	1546 (37.8)	699 (41.7)	621 (39.9)	55 (34.8)	51 (23.1)^c^	120 (24.8)^d^	
Tibetan	691 (16.9)	243 (14.50)	262 (16.9)	42 (26.6)^b^	35 (15.8)	109 (22.6)^d^	
Qiang	862 (21.1)	449 (26.8)	264 (17.0)^a^	6 (3.8)^b^	69 (31.2)	74 (15.3)^d^	
Others	994 (24.3)	285 (17.0)	408 (26.2)^a^	55 (34.8)^b^	66 (29.9)^c^	180 (37.3)^d^	
Education (n, %)							< 0.001
Illiterate	1381 (33.7)	409 (24.4)	479 (30.8)^a^	54 (34.2)	140 (63.4)^c^	299 (61.1)^d^	
Primary school	1563 (38.2)	684 (40.8)	655 (42.1)^a^	55 (34.8)	54 (24.4)^c^	115 (23.9)^d^	
Secondary school and above	1149 (28.1)	583 (34.8)	421 (27.1)	49 (31.0)	27 (12.2)^c^	69 (14.3)^d^	
Marital status (n, %)							< 0.001
Married	3223 (78.7)	1398 (83.4)	1224 (78.7)^a^	106 (67.1)^b^	175 (79.2)	320 (66.3)^d^	
single	870 (21.3)	278 (16.6)	331 (21.3)^a^	52 (32.9)^b^	46 (20.8)	163 (33.7)^d^	
Smoking history (n, %)							< 0.001
No	3209 (78.8)	1290 (77.2)	1191 (77.1)	123 (78.3)	192 (87.3)^c^	413 (86.0)^d^	
Yes	863 (21.3)	381 (22.8)	353 (22.9)	34 (21.7)	28 (12.7)^c^	67 (14.0)^d^	
Drinking history (n, %)							< 0.001
No	2973 (72.7)	1160 (69.2)	1129 (72.7)^a^	127 (80.4)^b^	163 (73.8)	394 (81.6)^d^	
Yes	1119 (27.4)	516 (30.8)	425 (27.3)^a^	31 (19.6)^b^	58 (26.2)	89 (18.4)^d^	
Number of chronic disease (n, %)							< 0.001
0	2099 (51.3)	922 (55.0)	750 (48.2)^a^	67 (42.4)^b^	120 (54.3)	240 (49.7)^d^	
1	897 (21.9)	386 (23.0)	342 (22.0)	27 (17.1)	50 (22.6)	92 (19.1)	
≥ 2	1097 (26.8)	368 (22.0)	463 (30.0)^a^	64 (40.5)^b^	51 (23.1)	151 (31.3)^d^	
Depression (n, %)							< 0.001
No	3292 (80.4)	1449 (86.5)	1257 (80.8)^a^	114 (72.2)^b^	170 (76.9)^c^	302 (62.5)^d^	
Yes	800 (19.6)	226 (13.5)	298 (19.2)^a^	44 (27.8)^b^	51 (23.1)^c^	181 (37.5)^d^	
Sleep duration (n, %)							< 0.001
< 6 h	485 (11.9)	189 (11.2)	209 (13.4)	20 (12.7)	20 (9.1)	47 (9.7)	
6–8 h	2698 (66.0)	1175 (70.1)	1014 (65.2)^a^	93 (58.9)^b^	142 (64.3)	274 (56.7)^d^	
≥ 9 h	910 (22.2)	312 (18.6)	332 (21.4)	45 (28.5)^b^	59 (26.7)^c^	162 (33.5)^d^	

^*^ Data are presented as the mean ± standard deviation (SD)

^a^ *P* values were < 0.05 between robust group and physical pre-frailty group

^b^ *P* values were < 0.05 between robust group and physical frailty group

^c^ *P* values were < 0.05 between robust group and cognitive impairment group

^d^ *P* values were < 0.05 between robust group and cognitive frailty group

Table 1 presents the characteristics of the study participants by physical frailty and cognitive impairment status. Significant differences were observed among the 5 groups regarding age, gender, ethnicity, education level, marital status, smoking history, drinking history, number of chronic diseases, depression and sleep duration (*P* <  0.001). Compared to other groups, cognitive frailty participants were more likely to be older (> 80 years), Tibetan, illiterate and single. The cognitive frail group contained a smaller proportion of participants with drinking history than other 4 groups. The proportion of participants who had high number of chronic disease (≥ 2), depression and long sleep duration (≥ 9 h) increased with the following order: robust, physically pre-frailty, physically frailty, cognitive impairment and cognitive frailty.

Table 2 shows the results of logistic regression analysis examining the association between frailty, cognitive function and sleep duration. It showed that long sleep duration (≥ 9 h) were significantly associated with an increased prevalence of pre-physical frailty (OR = 1.23, 95%CI = 1.03–1.47, *P* = 0.02), physical frailty (OR = 1.82, 95%CI = 1.25–2.66, *P* = 0.002), cognitive impairment (OR = 1.56, 95%CI = 1.13–2.17, *P* = 0.008) and cognitive frailty (OR = 2.23, 95%CI = 1.77–2.80, *P* <  0.001). Multivariate adjustment for age, sex, race, marital status, education level, smoking history, drinking history, number of chronic diseases and depression only resulted in small change to estimated OR in long sleep duration (≥ 9 h). Long sleep duration was independently associated with the increased odds of pre-physical frailty (OR = 1.20, 95%CI = 1.0–1.44, *P* = 0.049), physical frailty (OR = 1.70, 95%CI = 1.14–2.54, *P* = 0.009), cognitive impairment (OR = 1.45, 95%CI = 1.03–2.04, *P* = 0.032) and cognitive frailty (OR = 2.07, 95%CI = 1.60–2.68, *P* <  0.001) than the control group. Opposed to long sleep duration, the associations between short sleep duration (< 6 h) and cognitive frailty were not observed in either unadjusted or adjusted model.

**Table 2:** Associations between sleep duration and cognitive frailty according to unadjusted and adjusted logistic regression models (*n* = 4093)

	Robust	Physical pre-frailty OR [95%CI], *P*-value	Physical frailty OR [95%CI], *P*-value	Cognitive impairment OR [95%CI], *P*-value	Cognitive frailty OR [95%CI], *P*-value
Unadjusted model					
Sleep duration < 6 h	Reference	1.28 [1.03, 1.59], *P* = 0.023	1.33 [0.81, 2.22], *P* = 0.262	0.88 [0.54, 1.43], *P* = 0.597	1.07 [0.75, 1.51], *P* = 0.715
Sleep duration 6–8 h	Reference	Reference	Reference	Reference	Reference
Sleep duration ≥9 h	Reference	1.23 [1.03, 1.47], *P* = 0.020	1.82 [1.25, 2.66], *P* = 0.002	1.56 [1.13, 2.17], *P* = 0.008	2.23 [1.77, 2.80], *P* < 0.001
Adjusted model^a^					
Sleep duration < 6 h	Reference	1.19 [0.95, 1.48], *P* = 0.137	1.06 [0.62, 1.82], *P* = 0.824	0.82 [0.49, 1.36], *P* = 0.438	0.88 [0.60, 1.28], *P* = 0.499
Sleep duration 6–8 h	Reference	Reference	Reference	Reference	Reference
Sleep duration ≥9 h	Reference	1.20 [1.0, 1.44], *P* = 0.049	1.70 [1.14, 2.54], *P* = 0.009	1.45 [1.03, 2.04], *P* = 0.032	2.07 [1.60, 2.68], *P* < 0.001

^a^ Model adjusted for age, gender, ethnicity, marital status, education, smoking history, drinking history, number of chronic diseases, depression. *OR* odds ratio; *CI* confidence intervals

## Discussion

This is the first study to explore the association between cognitive frailty and sleep duration in a relatively large sample size of older adults in western China. Our results revealed that long sleep duration was associated with 2.07 times increased odds of cognitive frailty.

The overall prevalence of cognitive frailty in older adults dwelling in western China communities was 11.8%, which was generally consistent with the previous study conducted by Feng et al. [4]. They reported that the prevalence of pre-physical frailty and physical frailty concurrent with cognitive impairment was 10.7% in 2375 Chinese Singaporeans aged 55 years and older [4]. It is worth noting that the co-occurrence of pre-physical frailty, physical frailty and cognitive impairment were very common in our study. We found that 19.05% of physically pre-frail and 42.55% of physically frail participants had impaired cognitive function. The results were in line with previous studies that showed a higher prevalence of cognitive impairment in the older individuals with physical frailty [30–32]. Meanwhile, 51.99 and 16.62% of cognitive impaired participants had pre-physical frailty and physical frailty, respectively.

Results from our study indicated that long sleep duration was positively associated with pre-physical frailty, physical frailty, cognitive impairment and cognitive frailty. What’s more, higher odds risk of cognitive frailty was observed in the participants with long sleep duration among 5 groups. Although there is no direct research about the association between cognitive frailty and long sleep, several studies have found that physical frailty and cognitive impairment were related to long sleep separately, leading support to the link between cognitive frailty and long sleep duration. Sun et al. found that sleep time 9 h was associated with higher odds of physical frailty and pre-physical frailty in community-dwelling older adults aged 70–87 years [33]. Suh et al. demonstrated that older adults with long sleep duration (≥ 7.95 h) at baseline were related to the risk of cognitive decline at 4-year follow-up [34]. In addition, a prospective study focusing on frail elderly participants who were followed up for 18 months found that longer nighttime in bed increased the incidence of cognitive decline. These findings implied that long sleep duration can not only impair the physiological reserves of multiple systems in older adults, but also impair their cognitive function, thus ultimately leads to cognitive frailty.

Although exact mechanisms remain unclear, there are several similar underlying mechanisms between frailty and cognitive impairment and long sleep duration. Firstly, long sleep is known to be associated with cardiovascular disease, stroke, obesity and depression [35, 36]. It suggested that long sleep may be markers for poor health which increases the likelihood of frailty [37–40] and cognitive impairment [41–43]. Although we adjusted the number of diseases covariates and depression symptoms in this study, we could not ignore the possibility. Secondly, increased levels of pro-inflammatory factors C-reactive protein (CRP) and interleukin-6 (IL-6) were reported in participants with long sleep duration [44]. Meanwhile, the meta-analysis conducted by Soysal et al. indicated a positive association between physical frailty and pro-inflammatory factors, such as CRP and IL-6 [45], which was also found to be elevated in the participants with cognitive impairment [46, 47]. Therefore, inflammation is considered as one of the pathogenesis for physical frailty and cognition and long sleep duration. Furthermore, the atrophy of superior frontal gyrus (SFG) could be another possible underlying mechanism. Previous studies reported that SFG was involved in a variety of cognitive and motor control tasks [48–51]. A longitudinal study found those reporting > 7 h of sleep had higher rates of thinning in the SFG of the left hemisphere during an approximately 8-year follow-up period compared to those reporting 7 h of sleep [52]. These findings suggest the atrophy of SFG may increase the vulnerability of older adults for both physical and cognitive disorders through long sleep.

The present study did not observe the relationship between short sleep duration and frailty as well as cognitive impairment in older adults. Yet, previous studies have investigated the relationship among short sleep duration, frailty and cognitive function with inconsistent results. Similar to our results, both Sun et al. and Baniak et al. found no association between short sleep duration and frailty in either Americans or Chinese older adults [33, 53]. Tamayo et al. found sleeping less than 5 h could promote frailty in Mexican older women [17], while Ensrud et al. found no significant association between short sleep duration (< 5 h) and greater frailty status in American older men [54]. Meanwhile, one study in Korea demonstrated that short sleep duration (< 6 h) was not associated with frailty among older adults of both sexes [55]. Besides, a recent system review including 32 observational studies found that more studies suggested that long (rather than short) sleep duration were related to worse cognition [56]. The inconsistent results from different studies may be due to the differences of sleep duration’s definition (Night-time sleep duration or 24-Hour sleep duration) and short sleep duration’s classification (i.e., short sleep duration defined as < 4 h, < 5 h or < 6 h or < 7 h) [56]. The inconclusive results between studies may also raise the question of whether the older adults in different countries or regions have different sleep need.

The recurrent findings might provide a new approach for the prevention of cognitive frailty in older adults. Comparing to other risk factors, long sleep duration can be easily targeted in interventions by the geriatricians. Therefore, it is critical to evaluate sleep duration in the participants with cognitive frailty. Meanwhile, more intervention studies are needed to verify whether improving sleep duration is helpful for preventing or delaying cognitive frailty in the future. Nevertheless, some limitations of the present study need to be mentioned. First, similar to other cross-sectional studies, the causal association between sleep duration and cognitive frailty could not be established. In future research, longitudinal research should be conducted to verify this relationship. Second, sleep duration was only measured with self-reported questionnaire in this study. Although the application of self-reported questionnaire to assess participants’ sleep duration is common in routine clinical practice, self-reported sleep duration may introduce bias, especially when participants have severe cognitive impairment. Furthermore, there remains residual confounding factors in our study although we adjusted with many confounders in our study.

## Conclusions

In summary, our findings indicated that long sleep duration was an independent associated factor for cognitive frailty in older adults. As sleep duration is potentially modifiable, interventions directed to improve sleep may provide new strategies for the prevention of cognitive frailty in the future.

## Data Availability

The datasets used during the current study are available from the corresponding author on reasonable request.

## Abbreviations

AD: Alzheimer disease
BMI: Body mass index
CI: Confidence intervals
CIND: Cognitive impairment but no dementia
CLTPAQ: China leisure time physical activity questionnaire
CRP: C-Reactive protein
GDS-15: 15-Item geriatric depression scale
IL-6: Interleukin-6
IQR: Interquartile range
MLTPAQ: Minnesota leisure time physical activity questionnaire
OR: Odds ratio
QOL: Quality of life
SD: Standard deviation
SFG: Superior frontal gyrus
SPMSQ: Short portable mental status questionnaire
WCHAT: West China health and aging trend study
